# The prevalence of liquid chromatography-tandem mass spectrometry confirmed paediatric poisoning at Red Cross War Memorial Children’s Hospital, Cape Town, South Africa

**DOI:** 10.1186/s12887-021-02500-x

**Published:** 2021-01-18

**Authors:** Norbertta Washaya, Alicia Evans, Rudzani Muloiwa, Peter Smith, Heloise Buys

**Affiliations:** 1 Division of Ambulatory and Emergency Paediatrics, Red Cross War Memorial Children’s Hospital, Klipfontein Road, Rondebosch, Cape Town, 7700 South Africa; 2 Department of Paediatrics and Child Health, University of Cape Town, Cape Town, South Africa; 3 Division of Clinical Pharmacology, University of Cape Town, Cape Town, South Africa; 4 Groote Schuur Hospital, Cape Town, South Africa; 5 Red Cross War Memorial Children’s Hospital, Cape Town, South Africa

**Keywords:** Poisoning, Africa, Children, Mass spectrometry, LC-MS/MS toxicology results in poisoning cases

## Abstract

**Background:**

Paediatric poisoning is a common presentation to emergency departments worldwide. There is a paucity of data on the role of liquid chromatography-tandem mass spectrometry (LC-MS/MS), in the management of paediatric poisoning in low-and middle-income countries (LMICs). In high-income countries, most studies are retrospective, and few include children.

**Objective:**

The study describes the prevalence of liquid chromatography-tandem mass spectrometry confirmed paediatric poisoning at Red Cross War Memorial Children’s Hospital, Cape Town, South Africa.

**Methods:**

Children admitted with suspected poisoning between 1 January 2017 and 31 December 2017, were recruited. All patients had a urine and/or blood sample sent for LC-MS/MS toxicology. Data collected included demographic data, clinical features, investigations, management, outcome and social interventions.

**Results:**

One hundred fifty-two children, with median age of 39 (IQR 25–61) months were enrolled of which 128 (84%) were poisoning cases. Of the 128 poisoning cases, 88 (69%) presented with a history of ingesting a known substance, 16 (12%) an unknown substance and 24 (19%) were cases of occult poisoning. LC-MS/MS was able to identify a substance in 92% of the cases of occult poisoning. In those who had presented with a seemingly known substance, LC-MS/MS found a different substance in 15 cases. LC-MS/MS was also able to detect multiple drugs in 40 patients. Of the poisoning cases, six (5%) cases were attempted homicide cases and 5 (4%) cases were attempted suicide cases. No children died. Individualized social interventions were instituted in poisoning cases. Emergency placement safety reasons was required in 6 children.

**Conclusion:**

When the limitations are known, LC-MS/MS is useful in identifying cases of occult poisoning, identifying patients who have ingested multiple substances and/or an unknown substance and when targeted towards child protection. As LC-MS/MS is an expensive test, it should be used judiciously in LMICs.

## “Key messages” box

### Section 1: what is already known on this subject

The use of investigations in paediatric poisoning is controversial. There is a paucity of prospective data on the use of LC-MS/MS in paediatric poisoning in LMICS, where resources are constrained and risk factors for poisoning such as neglect and child abuse are high.

### Section 2: what this study adds

LC-MS/MS is beneficial in the paediatric patient who presents with occult poisoning or has ingested multiple and/or unknown substances. Requesting clinicians need to be aware of its shortfalls. In high risk settings, it can be utilized in community toxicovigilance and child protection. Due to its expense, a protocol needs to be developed for its judicial use in LMICS.

## Background

Paediatric poisoning is a common presentation to emergency departments worldwide [1, 2]. Though it has a good prognosis, it is an important cause of both morbidity and mortality [1, 3]. In 2016 it was responsible for 6,268,554 disability adjusted life years (DALYs) globally, with children less than 15 years accounting for 45% of these DALYs [4]. In a study done in South Africa, poisoning was responsible for 5.7% of all hospital admissions [2]. While, a retrospective patient folder review carried out at a hospital in Johannesburg, indicated that toxin ingestion was responsible for 17% of the admissions in to the paediatric intensive care unit [5].

In 2016, poisoning resulted in 31,400 unintentional deaths globally in children less than 15 years of age [6]. The death rate of poisoning was higher in low-and middle income countries (LMICs), with LMICs accounting for 69% of the deaths that year [6]. Despite the higher death rates in LMICs, data on the incidence of paediatric poisoning is more accurate in high-income countries (HICs) where poison control centers have been established and poisoning registries are kept [1].

Risk factors for poisoning include age, sex and environmental factors such as neglect [3, 7]. Child abuse, in particular neglect, is a big problem in low resource settings such as in Africa, especially in the under-5 population [8–11]. This under-5 population is the age group with the highest incidence of poisoning [1–3, 7, 12–14]. In LMICs, neglect may present as accidental poisoning as children are often left unsupervised, while child abuse may present as intentional or occult poisoning [15–17].

The role of investigations in poisoning is controversial but may be of benefit in occult poisoning, where it is difficult to confirm the presence and cause of poisoning. Point-of-care urine drug screen (POCUDS) testing is cost effective and readily available, able to give immediate results but has several disadvantages, such as, a high false positive rate; can only screen for a limited number of drugs; inability to quantify the drug; inability to name the drug, as it can only identify the drug class and the risk of false negatives if the drug in question is below the threshold cut-off for detection [18–22].

Liquid chromatography tandem mass spectrometry (LC-MS/MS) on the other hand, is a good confirmatory test [18, 20]. Unlike POCUDS, it has a higher sensitivity and specificity and has other advantages, such as, the increased breadth of substances that it can detect and its ability to identify and quantify drugs and their metabolites by name, and not just by the drug class [18, 21, 23, 24]. The main problem with LC-MS/MS, however, is that it is expensive and may have a long turnaround time [18, 19, 21].

Most of the studies done on the use of LC-MS/MS in poisoning have been done in a retrospective manner and in high-income settings [21]. Additionally, few of these studies have included children. The role of LC-MS/MS in LMICs, where the number of cases of child abuse and neglect are high and the resources to manage poisoned children are severely constrained, is not clear. Its use may be able to assist in identifying high-risk children in households that need social (child protection) interventions.

This study aims to describe the prevalence of LC-MS/MS confirmed poisoning in children who presented to a LMIC paediatric tertiary hospital over a period of a year, with an emphasis on the value that LC-MS/MS adds in LMICs.

## Methods

### Setting

The study was done at Red Cross War Memorial Children’s Hospital (RCWMCH), a public children’s hospital that provides secondary and tertiary health care services to children less than 13 years, living in urban, peri-urban and informal settlements. The hospital manages approximately 35,000 non-trauma emergency care patient-visits each year. A substantial proportion of the patients come from extremely poor and marginalized communities [25]. The children in the catchment area of RCWMCH are not only vulnerable because of poverty but also because of the increase in substance abuse in formal and informal settlements in the Western Cape Province of South Africa [8, 26, 27].

### Study design

The study prospectively enrolled patients with suspected poisoning admitted to the RCWMCH from the 1st of January 2017 to the 31st of December 2017, in a cross-sectional design.

### Participants

All patients admitted at RCWMCH with suspected poisoning were eligible for recruitment into the study if their legal guardians were willing to sign consent for them to be included. Patients who ingested corrosives requiring surgical intervention were excluded from the study.

### Data collection and procedures

After consent, data on demographic information, clinical presentation and results of investigations done by the attending clinician were taken, history was taken from the caregiver to establish possible causes of poisoning. The patient was followed up over the period of admission and management including clinical outcomes was recorded. The Poisoning Severity Score (PSS) was used to grade the severity of poisoning at admission (Table 1) [28].

**Table 1:** PSS grading

Grade	Description
None	No symptoms or signs related to poisoning
Minor	Mild, transient and spontaneously resolving symptoms
Moderate	Pronounced or prolonged symptoms
Severe	Severe or life-threatening symptoms
Fatal	Death

*PSS* Poisoning severity score. From Hans E Persson et al., 1998, Poisoning Severity Score. Grading of Acute Poisoning

### Toxicology investigation

A urine sample from eligible participants was sent to the laboratory for LC-MS/MS to establish the cause of poisoning. In addition, the attending clinician and laboratory were consulted for any leftover blood specimen after laboratory tests ordered by the attending clinician were completed that could likewise be tested on LC-MS/MS. Study participants were not bled solely for the study.

The LC-MS/MS unit used for this study was the, AB Sciex 3200 QTRAP (© 2013 AB Sciex Pty. Ltd., AB Sciex, 500 Old Connecticut Path, Framingham MA 01701–4574) unit, housed in the Division of Clinical Pharmacology, University of Cape Town, Groote Schuur Hospital, Cape Town. At the time of the study it had a library of 120 prescription drugs, over the counter medicines, illicit drugs and some of their metabolites. The library did not include pesticides or herbal compounds used in traditional medicines.

Due to the limited availability of the LC-MS/MS unit, samples were tested in batches. Once collected, samples were registered and transported to the laboratory where they were stored at 4 °C until analysis. The median turnaround time (TAT) for obtaining a result was 5 (interquartile range, (IQR) 3–7) days for urine LC-MS/MS and 6 (IQR 4–7) days for blood LC-MS/MS. A total of five patients had LC-MS/MS results within 24 h.

Trained personnel ran the samples and interpreted the results. For quality control, internal standards were added to each sample as part of the sample preparation. Each run included blanks, as well as positive and negative controls to ensure accurate results [29, 30].

In order to observe for possible substance degradation, compound stability tests were done on the LC-MS/MS unit. A commercially obtained control, a system suitability test (SST) (Restek® Corporation) was run daily. The kit contains 8 compounds of known concentrations. The peak areas of each compound were observed to confirm that the instrument performance and sensitivity were optimal and at the same time to observe for possible compound degradation, by comparing these areas to previously acquired data.

As poisoning is defined by the presence of clinical (somatic and/or mental) manifestations, or laboratory and/or electrocardiographic abnormalities resulting from exposure to a substance that can lead to harmful clinical effects [31], once all the toxicology investigations and clinical presentations were analysed, the authors classified the cases into one of three groups: substance-intake-unlikely, substance-intake-likely or substance-intake- unclear. The substance-intake-unlikely group were patients whose clinical presentation could be explained by an alternative medical diagnosis and were, therefore, not considered poisoning cases even though they were admitted as cases of suspected poisoning. The substance-intake-unclear group were patients whose clinical presentation could not be explained by a medical diagnosis and whose toxicology investigation results were not indicative of poisoning. The substance-intake-likely group were those patients whose clinical presentation could be explained by a toxic substance (even in the absence of an LC-MS/MS identified substance) and were therefore considered poisoning cases, even in the absence of symptoms.

Irrespective of laboratory results, for the purposes of our study, poisoning cases were also clinically divided by the authors into three groups: substance known (history of exposure to a known substance), substance unknown (history of exposure to an unknown substance) and occult poisoning (no history of poisoning, but clinical presentation in keeping with poisoning).

### Data analysis

Statistical analyses were done using STATA® 14.0 (StataCorp LLC, College Station, Texas, USA). The demographic characteristics and clinical findings at presentation were tabulated to provide a background description of the study population. All substances that tested positive with LC-MS/MS were described. Percentages and their 95% confidence intervals in outcomes of interest were used to depict proportions of categorical variables while medians with interquartile ranges were used to summarise continuous variables. The χ2 test or Fisher’s exact test were used to assess the strength of association between two categorical variables as appropriate. A significance level at a two-tailed *P* < 0.05 was used for all analyses.

## Results

### Demographic data

**Fig. 1:**
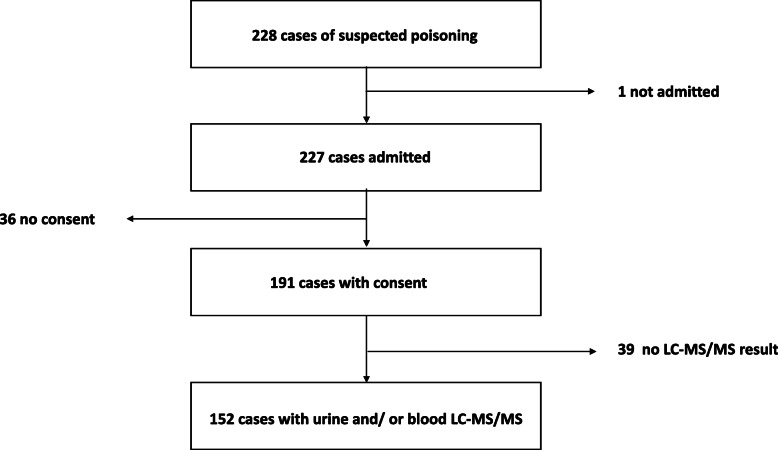
Study participant flow chart. LC-MS/MS- liquid chromatography tandem mass spectrometry

A total of 228 cases of suspected poisoning were screened of which 152 were included (Fig. 1). The median age of the included children was 39 (IQR 25–61) months, of whom 86 (56%) were male and 113 (74%) were below 5-years-of age (Table 2).

**Table 2:** Baseline characteristics of the study population (*N* = 152)

Variable		n (%)
Male		86 (56%)
Age	< 1 year	14 (9%)
	1–5 years	99 (65%)
	> 5–12 years	31 (21%)
	> 12 years	8 (5%)
Housing	Formal	96 (63%)
	Informal	39 (26%)
	Unknown	17 (11%)

### Toxicology results

A total of 146 (96%) urine samples from the 152 study participants were analysed by LC-MS/MS after six samples were lost due to leakage in transit. For 80 (53%) participants, there was sufficient left-over blood specimen in the laboratory for LC-MS/MS testing. This included the six participants whose urine samples had been lost to leakage. Altogether, in 89/152 (59%) participants a substance was detected. In 16 (18%) of these the detected substances were iatrogenic secondary to administration of in-hospital care or therapy given at home. After discounting the iatrogenic substances or medicines given at home 73 of 152 (48, 95% CI 40–56%) participants had a substance detected by LC-MS/MS.

In total, 128 (84%) of the children, 71 (55%) in whom a substance was detected on LC-MS/MS, were classified as genuine cases of poisoning (substance-intake-likely), while 15 (10%) of the 152 were classified as unlikely to have been poisoned (substance-intake-unlikely). In nine (6%) of the children it was not clear whether poisoning had taken place or not (substance-intake-unclear).

Despite being classified as genuine cases of poisoning, 57 (45%) of the 128 substance-intake-likely children did not have a causative substance identified via LC-MS/MS and 49 (38%) children had no substance identified and eight (6%) had iatrogenic substances identified. The median TAT for the 57, who were substance-intake-likely cases but in whom the LC-MS/MS was negative, was 5 (IQR 3–9) days for urine LC-MS/MS and 5 (IQR 4–9) days for blood. TAT of the 71 poisoning cases that had positive LC-MS/MS was 5 (IQR 2–7) days for urine and 6 (IQR 4–7) days for blood.

In 26 (20%) of the substance-intake-likely group in whom no substance was detected, the suspected substance was not in the LC-MS/MS reference library used. Of these, 17/26 (65%) were pesticides (11 rat ‘poison’, 4 ‘cockroach poison’, 1 ‘tick poison’ and 1 undefined pesticide).

There were eight organophosphate poisonings cases in the substance-intake-likely group. In two of the eight organophosphate poisonings, LC-MS/MS detected other substances (bromazepam and diphenhydramine), ingested by the same patients. Likewise, in one of the four cases of iron poisonings, trimethoprim was concomitantly identified by LC-MS/MS. Eight patients who had ingested hydrocarbons, three ethanol ingestions, two turpentine, and one each of petrol, eucalyptus oil and paraffin ingestion, had no additional substances detected by LC-MS/MS.

Five (4%) patients in the substance-intake-likely group presented with a history of ingesting an unknown substance, and the identity of the unknown substance was not identified via LC-MS/MS. Cannabis was detected via LC-MS/MS in a tablet brought by one of these patients but could not be confirmed in the patient’s samples.

Nine patients in the substance-intake-likely group presented after ingesting a substance found in the LC-MS/MS library and yet the substance was not detected by LC-MS/MS, despite seven patients being symptomatic from the suspected substance. Four of the nine patients had both blood and urine LC-MS/MS done, while five had only urine LC-MS/MS done. The drugs that were not detected were the following, clonazepam, diazepam, lorazepam, phenytoin, alprazolam, cannabis, antiretrovirals (tenofovir/emtricitabine/efavirenz), chlorpromazine and tricyclic antidepressant. Six of the patients had vomiting induced by the care giver in an attempt to decontaminate. Furthermore, two of these patients received charcoal before the LC-MS/MS was done (one case of tricyclic antidepressant toxicity and one case of chlorpromazine ingestion). The median TAT for these nine patients was 7 days with a range of 1–13 days.

Of the 15 patients, in the substance-intake-unlikely group, LC-MS/MS detected no substances in eight (53%) and identified iatrogenic medicines in seven (47%). Of the nine substance-intake-unclear patients, one patient had a positive result due to iatrogenic medicines and two had positive results, but the drugs identified could not explain the clinical presentation.

### Presenting history versus LC-MS/MS results in poisoning cases (substance-intake-likely)

**Fig. 2:**
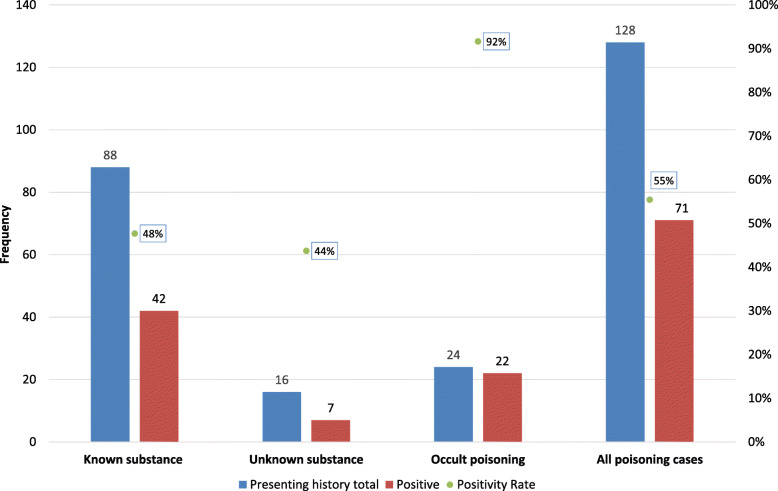
Number and proportion of substance detection rates on LC-MS/MS in substance-intake-likely group. LC-MS/MS liquid chromatography tandem mass spectrometry

When the 128 children in the substance-intake-likely group was further analysed according to the history obtained from the caregiver, 24 (19%) participants had no history of exposure to a substance (occult poisoning). (Fig. 2) In those who had occult poisoning, the suspicion of poisoning came from the clinician’s examination findings, and/or investigations done by the attending clinician. The substance detection rate of LC-MS/MS, after removing iatrogenic medicines, was then analysed in three different groups, known substance, unknown substance and occult poisoning. (Fig. 2).

In children with occult poisoning, LC-MS/MS was able to identify the substance in 22/24 (92%) compared to 42/88 (48%) when a guardian reported ingestion of a known substance (*p* = < 0.0001), and 7/16 (44%) when a guardian reported ingestion of an unknown substance (*p* value = 0.003) (Fig. 2).

In the 22 (92%) cases of occult poisoning, in which LC-MS/MS identified a substance, the substance identified was in keeping with the clinical presentation in 20/22 (91%). The two patients, in whom LC-MS/MS identified a substance not in keeping with the clinical presentation, concomitant organophosphate poisoning was identified by alternative means. In these two organophosphate cases LC-MS/MS identified a substance that would have otherwise been missed. All 15 patients who had presented with an unknown substance and 23 (96%) of the 24 cases of occult poisoning had neurological symptoms.

In the patients who reported ingesting a seemingly ‘known’ substance, the substance found on LC-MS/MS was different in 15/88 (17%) patients. In these 15 cases, six were asymptomatic, while four had symptoms consistent with the substance found on LC-MS/MS.

Overall, 18/128 (14%) cases of poisoning would have been missed had LC-MS/MS not been used in this study.

### Causes of poisoning

In 106/128 (83%) of the cases, poisoning was unintentional. There were however 6/128 (5%) cases of attempted homicide and 5/128 (4%) of attempted suicide (Table 3).

**Table 3:** Causes of poisoning (Intent), *n* = 128

Intention		Frequency (***N*** = 128)
Unintentional	Self	99 (77.3%)
	Caregiver medication error	1 (0.8%)
	Traditional medicine	3 (2.3%)
	Iatrogenic	3 (2.3%)
Intentional	Attempted homicide	6 (4.7%)
	Caregiver/adult but not attempted homicide	6 (4.7%)
	Attempted suicide	5 (3.9%)
	Self but not suicide attempt	1 (0.8%)
Undetermined		4 (3.1%)

Of the six attempted homicides, two cases involved siblings from a family that had three deaths due to the same organophosphate poisoning event. In one of the patients who had been given traditional medicines, norfluoxetine, trimethoprim and diphenhydramine were detected by LC-MS/MS. Four of six children given substances intentionally by adults received drugs of abuse- two received cannabis, one received methamphetamine and the other ethanol. The other two patients, presented with neurological symptoms, and the substances administered could not be identified.

### Drugs identified by LC-MS/MS

**Fig. 3:**
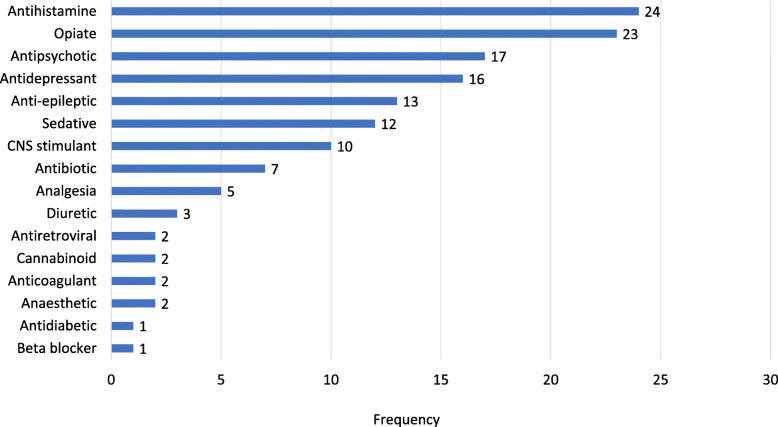
Drug classes identified by LC-MS/MS. LC-MS/MS: liquid chromatography tandem mass spectrometry

LC-MS/MS was able to identify a total 45 different drugs after removal of iatrogenic medicines and medicines given at home (Fig. 3). In the 128 substance-intake-likely cases, LC-MS/MS identified 140 substances. The most common causative group identified by LC-MS/MS was antihistamines found in 24 (19%) patients, followed by opiates in 23 (18%) and antipsychotics in 17 (13%). The most common drugs were chlorpheniramine and haloperidol found in 9 (7%) patients each. LC-MS/MS was able to identify multiple drugs in 40 (31%) of the substance-intake likely group.

### Comparison of urine and blood LC-MS/MS results

Seventy-four (74) patients had both urine and blood samples analysed on LC-MS/MS. Urine and blood LC-MS/MS yielded the exact same result in 48 (65%) patients (Table 4). In 18 (24%) of the participants with paired samples, more substances were detected in urine but not in blood, while in 4 (5%) samples, more substances were detected in blood but not urine.

**Table 4:** Comparing urine and blood LC-MS/MS positivity rate (*N* = 74)

LC-MS/MS Result	Frequency (%)
No detected substance in urine and blood	27 (36%)
Same substance detected in urine and blood	21 (28%)
Different substance detected in urine and blood	4 (5%)
Substance detected in urine and blood, but more substances found in urine	7 (9%)
Substance detected in urine and blood, but more substances found in blood	3 (4%)
Substance detected in urine but not in blood	11 (15%)
Substance detected in blood but not in urine	1 (1%)
**Total**	**74 (100%)**

*LC-MS/MS* Liquid chromatography tandem mass spectrometry

### Clinical systems involved in the poisoning cases

Of the 71 positive LC-MS/MS results in the substance-intake-likely group, the substances identified by LC-MS/MS were in keeping with the clinical presentation in 55/71 (77%) participants. Nine (13%) of the 71 positive LC-MS/MS cases in the substance- likely-group were asymptomatic even though a substance was detected by LC-MS/MS.

The most common system involved was neurological, found in 88 (69%) of the substance-intake-likely cases followed by gastrointestinal found in 49 (38%), cardiovascular in 26 (20%) and 22 (17%) were asymptomatic. Of the 49 that had gastrointestinal symptoms 24 (49%) had the presence of the confounder of intentional induction of vomiting by the caregivers using manual induction, milk and/or saltwater. LC-MS/MS detected a substance in 58 (66%) out of 88 poisoning cases with neurological symptoms compared to 13 (33%) of the 40 without neurological symptoms (*p* < 0.0001).

### Substance-intake-likely management and outcome

According to the PSS, most cases were classified as moderate, 51 (40%), while 12 (9%) were classified as none and 42 (33%) were minor and therefore required minimal supportive care. Of the 23 (18%) children with a PSS severe grade, 10 (8%) required admission to the Paediatric Intensive Care Unit (PICU). Twenty-nine children (23%) were given an antidote and 6 (5%) received activated charcoal. There were no deaths.

Individualized social intervention was instituted in all the patients with removal and emergency placement occurring in six patients. All six attempted homicide cases were referred for forensic investigation. The mother was the perpetrator in four of the attempted homicide cases. LC-MS/MS detected a substance in three of the attempted homicides. A total of 22 (14%) patients had an LC-MS/MS result prior to discharge.

## Discussion

Our study describes the prevalence of LC-MS/MS-confirmed paediatric poisoning in a LMIC setting. LC-MS/MS was particularly helpful in occult poisoning where it was able to identify over 90% of the substances, as well as in identifying multiple substance ingestion. In addition, our study indicates the urine sample as having a higher detection rate for identifying potential ingested substances when compared to a blood specimen. According to the authors’ knowledge, this study is the first prospective one of its kind done in a LMIC setting.

Similar to previous studies, most of the poisoning cases were males between the ages of one and 5 years [1–3, 7, 12–14]. This is likely due to the developmental stage toddlers are in, that involves curiosity about the world and a desire to explore it [13, 14].

Previous literature has demonstrated shortfalls with urine point of care drug screen immunoassay, therefore, a positive point of care urine drug screen result requires a confirmatory test, such as mass spectrometry [18, 20–22]. After excluding iatrogenic medicines and therapy given prehospital, LC-MS/MS was able to detect substances in 48% of all study participants and 55% in the substance-intake-likely cases.

Twenty-six patients in this study reported ingesting a substance that was not in the library. This was the main reason for a negative LC-MS/MS result in poisoning cases, in this study. This indicates that the ability of LC-MS/MS to detect a substance is limited by the extent of the LC-MS/MS library available at the time. Notably, the LC-MS/MS library can be updated and additional drugs/substances added [18, 19, 21]. The LC-MS/MS used in this study could detect the presence of various drugs in concentrations as low as 20 ng/ml. Despite this high sensitivity, nine poisoning cases who had ingested drugs in the LC-MS/MS library were not detected. The possible explanations are varied and include, that the concentration of these drugs in the analysed samples may have been below the limit of detection of the instrument, either due to rapid metabolism or elimination. Six of the nine patients had vomiting induced by the care givers which could have led to decontamination, before the patient could absorb the drug. Notably, two of these patients were given activated charcoal. Worryingly, the first was a tricyclic antidepressant overdose, that LC-MS/MS did not detect. The second was a symptomatic chlorpromazine ingestion. This ingestion was witnessed, and the patient was given activated charcoal before the LC-MS/MS was done. It is possible that LC-MS/MS may have been limited by failure to detect substances that are eliminated via the hepatobiliary system which may not have been detectable in urine, as well as substances with a short half-life that may have degraded before sampling or analysis. These reasons are limitations of LC-MS/MS that the clinician needs to be aware of when utilising LC-MS/MS. All nine drugs that the LC-MS/MS failed to identify, and yet were in the LC-MS/MS library, are excreted in urine except for tenofovir, which is mainly excreted in faeces. A study done on sample stability indicated that substance degradation was dependent upon the type of substance and the temperature at which a sample is stored [32]. Substances stored at 25 °C, 4 °C and − 20 °C were later extracted and analysed at 15, 60 and 90 days and the average relative peaks on these days were compared with the average relative peaks at baseline [32]. The study concluded that the best temperature to store samples is − 20 °C, although even at 4 °C the substances could still be detected even if the peaks were lower [32]. The samples in our study were stored at 4 °C, and therefore substance degradation cannot be ruled out.

There are other substances that the LC-MS/MS could not detect, and these included: volatile substances such as hydrocarbons, which require a different method i.e., gas mass spectrometry for detection; organophosphates because they metabolize fast and metals such as iron. As a result, though a positive LC-MS/MS result is beneficial, a negative LC-MS/MS result does not rule out poisoning.

Due to circumstantial evidence, such as an open bottle or missing tablets, the causative agent in paediatric poisoning is generally obtained from history, which means laboratory investigations to identify the cause of poisoning is often regarded as not necessary. However, in our study, LC-MS/MS found that of the 88 poisoning cases that had ingested a seemingly ‘known’ substance, almost a fifth (17%), of the patients had ingested a different drug from that reported by the caregiver. This has management implications as the wrong drug level can be requested from the laboratory and the wrong antidote given while the right one is delayed.

Most of the studies and reports that look at the causes of poisoning in children do not highlight multiple drug exposure as an important cause of poisoning [1, 7, 13, 33]. Veale et al., in a study that included both adults and children, indicated that only 13.8% of the poisoning cases had been exposed to multiple drugs [12]. While a retrospective study done at the same children’s hospital as our study setting, indicated that less than 10% of the children who presented with poisoning had been exposed to more than one toxin [3]. Contrary to the previous mentioned studies, that reported low rates of multiple drug ingestion in children, in our study, LC-MS/MS detected 40 (31%) cases of multiple drug ingestions further demonstrating the ability of LC-MS/MS in positively identifying multiple drug ingestions. The use of laboratory specific drug levels to detect multiple drugs requires the clinician to request different specific drug levels to be run, in contrast, LC-MS/MS requires only one sample to be run to identify multiple drugs and/or substances. Without LC-MS/MS multiple drug ingestions would have been missed in this cohort of children. However, it is important to note that LC-MS/MS was not able to differentiate between multiple drugs from a single medicine with two or more drugs, and that which involved ingestion of multiple separate drug formulations.

The most common drug classes found in our study were antihistamines (19%), opiates (18%), antipsychotics (13%) antidepressants (12%) and antiepileptics (10%), while the most common drugs detected on LC-MS/MS were chlorpheniramine and haloperidol. This may explain the high frequency (69%) of neurological symptoms in the cases with likely substance ingestion. Historically, agro-based and non-drug chemicals were the main causes of poisoning in LMICs [1, 3, 7, 33–35]. There is a need to strengthen preventative campaigns in LMICs as pharmaceuticals are becoming important causes of poisoning [12, 34].

Traditional medicine use is not uncommon in LMICs, there have been previous reports of these medicines being adulterated, as was the case in one of our patients who ingested traditional medicine and LC-MS/MS identified norfluoxetine, trimethoprim and diphenhydramine [24, 36–39].

While, both blood and urine samples can be analysed by LCMSMS, urine is usually readily available as a non-invasive specimen with minimal discomfort to children. Furthermore, unlike in blood, drugs and their metabolites are known to remain in urine for longer (up to 1 week) post last exposure depending on the drug [20, 21, 40]. This gives a greater window of opportunity to still identify a substance after ingestion, especially when this is unknown or occult.

It is important to note that the clinical outcome was not altered using LC-MS/MS, this corresponds to previous studies, and in our study was because of the long turnaround time, with a median of 5 (IQR 3–7) days for urine LC-MS/MS and 6 (IQR 4–7) days for blood [19, 21]. In our study, the turnaround time was prolonged because the test samples were batched. The other major limitation of mass spectrometry is its expense [18, 19]. However, as technology has improved, mass spectrometers have become cheaper and faster [18, 23, 29, 41–43]. A study by Caspar et al., demonstrated its value in 24/7 toxicology by analysing 22 drugs and active metabolites in a qualitative and quantitative manner [30]. In the study done by Caspar et al., the total run time for a test was 11 min, extrapolated to the emergency care setting, such run times would enable the clinician to treat the patient accordingly and in a timely point-of-care manner [30, 43]. It would also avoid unnecessary treatment procedures in those that do not require them. The LC-MS/MS system may also be made more efficient using automation, this reduces the need for skilled personnel to run the equipment [18, 21].

LC-MS/MS identified 92% of all cases of occult poisoning and the substance identified by LC-MS/MS were in keeping with the clinical presentation in 91% of the cases of occult poisoning. There is limited data on the prevalence of occult poisoning in children especially in LMICs, in our study, one in five (19%) of the poisoning cases were due to occult poisoning. Occult poisoning was more likely if the patient had acute unexplained neurological symptoms that were not due to an infection or trauma. This makes LC-MS/MS of value in the area of child protection, when children may be poisoned intentionally. Child protection is also required in all cases of unintentional poisoning that are due to neglect. In this study six children required removal from the adverse environment as well as further child protective measures.

## Limitations

Our study is limited by a small sample size which reduced our ability to stratify the data further by causes of poisoning. Furthermore, we were not able to include unnatural home deaths that presented to the mortuary. As alluded to earlier, the LC-MS/MS library used did not contain an exhaustive list of possible substances.

## Conclusion

In conclusion, the use of LC-MS/MS in toxicology screening is novel in the African paediatric population. It appears to be of greatest value in the paediatric patient who presents with occult poisoning or has ingested multiple and/or unknown substances. It was less helpful in those that had ingested a known single substance unless the substance found on LC-MS/MS was found to be different. Though a robust test, clinicians need to be aware of its shortfalls. In high-risk settings, it can be utilized in community toxicovigilance and child protection. Finally, the authors could not find guidelines on the use of investigations, in particular LC-MS/MS, in LMICs, and because LC-MS/MS is an expensive test, we recommend that a protocol for its judicial use in LMICs be developed.

## Data Availability

All the data that support the findings of this study are contained within the manuscript. Any requests for additional data can be made available upon reasonable request from the corresponding author and with permission of The Human Research and Ethics Department of the University of Cape Town.

## Abbreviations

DALYs: Disability adjusted life years
HICs: High-income countries
IQR: Interquartile range
LC-MS/MS: Liquid chromatography tandem mass spectrometry
LMICs: Low-and middle-income countries
PICU: Paediatric Intensive Care Unit
POCUDS: Point-of-care urine drug screen
PSS: Poisoning Severity Score
RCWMCH: Red Cross War Memorial Children’s Hospital
SST: System suitability test
TAT: Turnaround time
χ2 test: Chi-square test
